# Dual positive and negative control of *Chlamydomonas* PII signal transduction protein expression by nitrate/nitrite and NO via the components of nitric oxide cycle

**DOI:** 10.1186/s12870-018-1540-x

**Published:** 2018-11-27

**Authors:** Zhanneta Zalutskaya, Lidiya Kochemasova, Elena Ermilova

**Affiliations:** Biological Faculty, Saint-Petersburg State University, Saint-Petersburg, Russia

**Keywords:** *Chlamydomonas reinhardtii*, Nitrate, Nitrite, NO signaling, PII signal transduction protein, Truncated hemoglobin

## Abstract

**Background:**

The PII proteins constitute a large superfamily, present in all domains of life. Until now, PII proteins research in Chloroplastida (green algae and land plants) has mainly focused on post-translation regulation of these signal transductors. Emerging evidence suggests that PII level is tightly controlled with regard to the nitrogen source and the physiological state of cells.

**Result:**

Here we identify that a balance of positive (nitrate and nitrite) and negative (nitric oxide) signals regulates *Chlamydomonas GLB1*. We found that PII expression is downregulated by ammonium through a nitric oxide (NO)-dependent mechanism. We show that nitrate reductase (NR) and its partner, truncated hemoglobin 1 (THB1), participate in a signaling pathway for dual control of *GLB1* expression. Moreover, NO dependent guanilate cyclase appeared to be involved in the negative control of *GLB1* transcription.

**Conclusion:**

This study has revealed the existence of the complex *GLB1* control at transcription level, which is dependent on nitrogen source. Importantly, we found that *GLB1* gene expression pattern is very similar to that observed for nitrate assimilation genes, suggesting interconnecting/coordinating PII-dependent and nitrate assimilation pathways.

**Electronic supplementary material:**

The online version of this article (10.1186/s12870-018-1540-x) contains supplementary material, which is available to authorized users.

## Background

Inorganic nitrogen (N) acts as one of the most important mineral nutrients for all autotrophic organisms including plants. In natural ecosystems, the availability of nitrogen is often a limiting factor for plant growth. Plants have evolved highly efficient and selective systems for nitrogen acquisition to ensure an appropriate utilization of the scarce resources. In all domains of life [1–3] with representatives in most bacteria and in many archaea [4, 5] as well as in oxygenic eukaryotic phototrophs [6], regulation of N metabolism at various levels are coordinated by members of PII signal transduction proteins [7, 8]. PII proteins act as reporters of the metabolic state of the cell by interdependent binding of ATP/ADP and 2-oxoglutarate (2-OG) [9–11]. The conserved mode of PII function is based on the control of PII – target protein interactions via the effector molecules binding [12]. Furthermore, in plants, the cellular glutamine levels are additionally sensed via PII signaling [8, 12, 13].

A second, phylogenetically diverse regulatory mechanism is covalent modification of apical residues of the T loop in PII proteins that allows the integration of additional signals. In proteobacteria, actinobacteria and cyanobacteria PII proteins can be covalently modified by uridylylation, adenylylation and phosphorylation at the T loop residues, respectively [14–16].

However, in many other organisms, this second regulatory layer of covalent modification of the T loop is apparently missing, as in Archaea [7], *Bacillus* [17], and plant PII proteins [6, 18]. The lack of this regulatory level can be partially compensated by control of PII-encoding genes at transcription level [19]. In Chloroplastida (green algae and land plants) PII-encoding *GLB1* genes are nuclear-encoded and, in Rhodophyta they are coded by the plastid genome [6]. It is believed that regulation of PII in plants may be transcriptional [20–22]. However, unlike bacteria, the transcriptional control of plant PII expression remains poorly understood.

*Chlamydomonas reinhardtii* (*Chlamydomonas* in the following) is a model alga that shares with higher plants the capability of controlling by PII the activity of N-acetyl-L-glutamate kinase (NAGK) that leads to arginine formation [8]. Although *Chlamydomonas* efficiently uses nitrate and nitrite, ammonium is preferred N source and many genes involved in nitrate/nitrite assimilation are repressed in the presence of ammonium [23, 24]. It is also important to note that amino acids are extracellularly deaminated by *Chlamydomonas* and only ammonium enters the cells [25]. Given that ammonium depletion induces *Chlamydomonas GLB1* upregulation [21], we hypothesized that ammonium may play a role in such negative regulation and this gene may respond to a balance of negative and positive signals.

In *Chlamydomonas,* ammonium and nitric oxide (NO) inhibit the expression of high-affinity nitrate/nitrite transporters and nitrate reductase (NR) [24]. During the cycle NO_3_^−^ → NO_2_^−^ → NO→ NO_3_^−^ the negative signal of NO can be converted back to the positive signal of nitrate. Recent publications have uncovered the function of NR in this cycle [26]. NR acts as an essential partner protein of the nitric oxide-forming nitrite reductase (NOFNiR) that catalyzes the formation of NO from nitrite [27]. Furthermore, NR is a protein partner of truncated hemoglobin 1 (THB1) for the conversion of NO into nitrate [28]. In spite of the key role of nitrate as a major nutrient and signal molecule, its possible regulatory effects on *GLB1* transcription have not been analyzed. In order to understand the processes PII is involved in, it is important to know how *GLB1* gene expression is regulated and when the amount of this protein is increased.

This apparent gap in the information about plant PII control motivated us to investigate the role of the components of cycle NO_3_^−^ → NO_2_^−^ → NO →NO_3_^−^ in regulating *GLB1* transcription in *Chlamydomonas* cells. In this work, we unveil that *GLB1* expression responds to an extracellular NO_3_^−^/NH_4_^+^ balance. Moreover, we show that nitrate and nitrite induce *GLB1*, and NO represses this gene. Collectively, these results suggest that the NR and its partners, NOFNiR and THB1, participate in a signaling pathway for dual control of *GLB1* expression.

## Methods

### Algal strains, growth conditions and cell treatment

The following *Chlamydomonas reinhardtii* strains were used: wild-type cw15–325 (*mt+, cw15, arg7*), which was kindly provided by Dr. M. Schroda (University of Kaiserslautern, Germany) and transformants with reduced THB1 obtained from cw15–325 *ami*THB1–11 (*mt+, cw15*), *ami*THB1–14 (*mt+, cw15*) and *ami*THB1–23 (*mt+, cw15*) [29]. The 305 mutant (*mt*^*−*^*nit1)* affected in NAD(P) H-NR activity and without diaphorase-NR activity was originally obtained from the wild type 6145c (*mt*^*−*^) [30]. The 305 and 6145 strains were kindly provided by Dr. E. Fernández (University of Cόrdoba, Spain).

Cells were grown mixotrophically in tris-acetate-phosphate (TAP) medium (https://www.chlamycollection.org/methods/media-recipes/tap-and-tris-minimal/) containing 7.5 mM NH_4_Cl instead of NH_4_NO_3_ under continuous illumination with white light (fluence rate of 45 μmol m^− 2^ s^− 1^) at 22 °C with constant orbital agitation at 90 rpm. The TAP medium was supplemented with 100 mg L^− 1^ of arginine when required. Cells were collected at the midexponential phase of growth by centrifugation (4000 *g*, 5 min), washed twice with 10 mM potassium phosphate, pH 7, before being transferred to the induction media containing the different sources of nitrogen and chemicals. At each harvesting times the number of viable cells were counted microscopically with use of 0.05% (*v*/v) Evans blue (DIA-M, Russia) as described [31]. Non-viable (stained) and viable (unstained) cells were counted. Four-hundred cells from each sample were scored for three biological replicates.

Determination and calculations of total chlorophyll (μg/ml) were performed as previously described [29, 32].

The compounds DEA-NONOate [2-(*N*, *N*diethylamino)-diazenolate 2-oxide sodium salt] and ODQ [1H-(1,2,4])oxadiazolo(4,3-a) quinoxalin-1-one] are from Sigma-Aldrich.

### Gene expression analysis

The total RNA was isolated with Trizol according to the manufacturer’s instructions (Invitrogen, USA). To remove genomic DNA, the RNA samples were treated with RNase-Free DNase I (Fermentas). Subsequently, RNA concentration and purity (260/280 nm ratio) was determined using spectrophotometer (SmartSpec Plus, Bio-Rad).

Revert Aid HMinus First Strand cDNA Synthesis Kit (Thermo Scientific) was used for reverse transcription reaction. The primer pairs for RTqPCR are given in Additional file 1: Table S1. RT qPCR was performed with a CFX96 Real-Time PCR Detection System (Bio Rad) using SYBR Green I according to [33]. Gene expression ratios were calculated with the ΔΔCt method [34]. The *RACK1* (receptor of activated protein kinase C; Cre13.g599400) gene was chosen as the control housekeeping gene. All reactions were performed in triplicate with at least three biological replicates. Significant differences between experiments were evaluated statistically by standard deviation and Student’s t-test methods.

### Protein gel blot analysis

The protein content was determined with amido black staining and protein gel blot analysis was performed as described [33, 35]. After separation by SDS-PAGE on a 12% polyacrylamide gel (*w*/*v*), the proteins were transferred to nitrocellulose membranes (Carl Roth, Karlsruhe) with use of semidry blotting (Trans-blot SD BioRad). The dilutions of the primary antibodies used were as follows: 1:5,000 anti-CrPII and 1:2000 anti-HSP70B. As a secondary antibody, the horseradish peroxidase-conjugated anti-rabbit serum (Sigma) was used at a dilution of 1:10,000. The peroxidase activity was detected via an enhanced chemiluminescence assay (Roche). For quantification, films were scanned using Bio-Rad ChemiDocTMMP Imaging System, and signals were quantified using the Image LabTM software (version 5.1).

### Nitrate determination

After eliminating the cells by centrifugation at 3000 g, nitrate concentrations in the medium were determined by dual-wavelength ultraviolet spectrophotometry as A_220_ - 2A_275_ using standard curve [36]. For the measurements, media with 4 mM nitrate were diluted 50-fold. Values were obtained from at least three biological replicates; each replicate was analyzed three times. Student’s *t*-tests were used for statistical comparisons. *P*-values of< 0.05 were considered as significant.

### Measurement of NO

Cells were treated with DEA-NONOate or nitrite, then they were incubated with in the presence of 1 μM (4-amino-5-methylamino-2′7’-difluorofluorescein diacetate) dye (DAF-FM DA, Sigma-Aldrich), at concentration of 45 μg/ml chlorophyll. After 15 min the cells were washed, resuspended in indicated medium and used for the fluorometric detection of NO. The supernatant was collected in a test tube and then used to detect NO in the medium. The measurement of NO was carried out with a microplate reader CLARIOstar (BMG) as described [29]. The excitation and emission wavelengths for the NO indicator were 483 ± 14 and 530 ± 30 nm, respectively. Fluorescence intensity was calculated as arbitrary units per chlorophyll or protein as described previously [29].

### NO detection by confocal microscopy

Cells were treated as described above. Images were acquired with a Leica TCS-SP5 confocal microscope (Leica-Microsystems, Germany) as described [29]. All experiments were performed in triplicate.

## Results

### *GLB1* is induced by nitrate

**Fig. 1:**
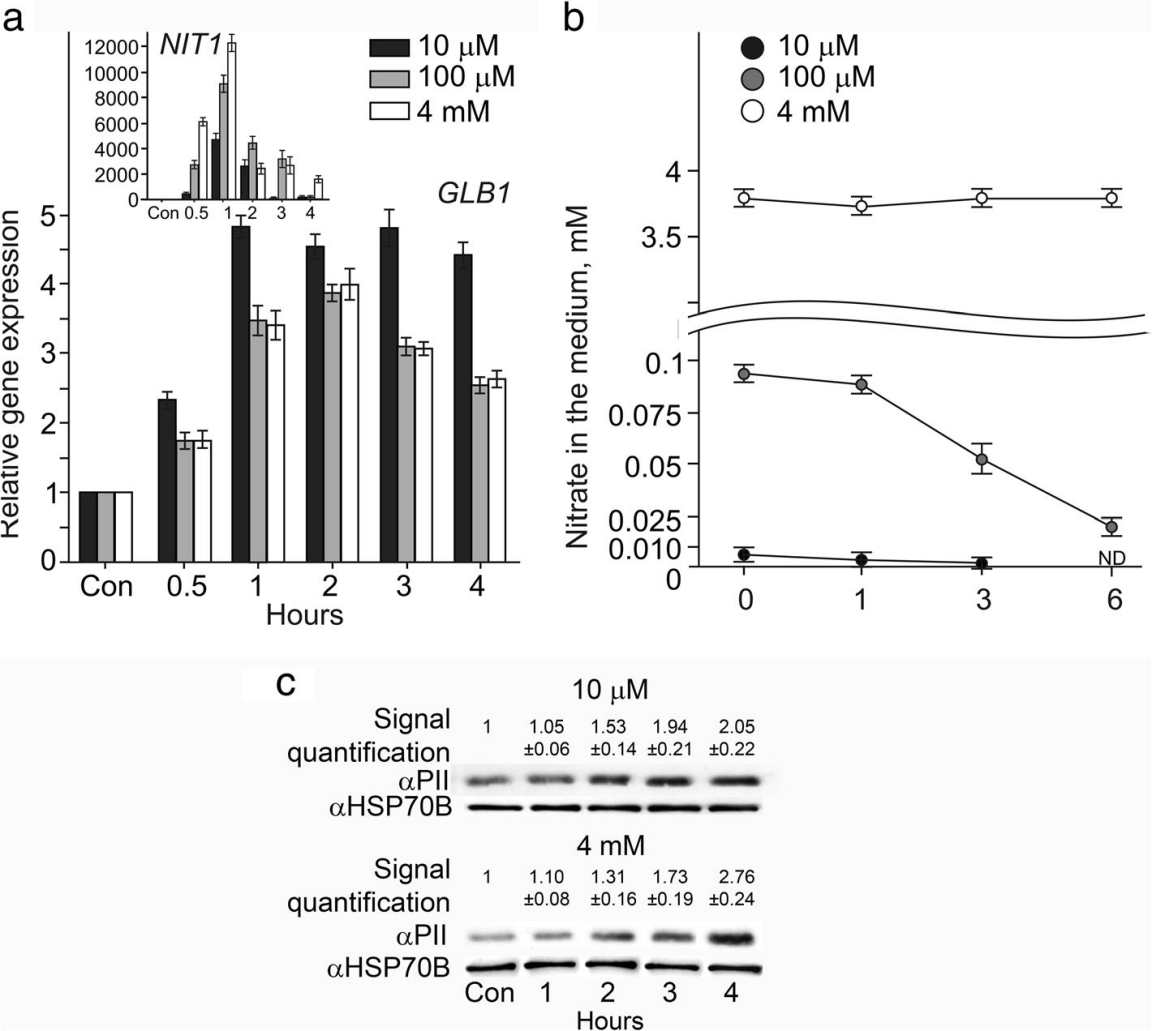
PII expression is increased by nitrate. **a** Time course of the *GLB1* transcripts accumulation during incubation of illuminated cells in nitrate-containing medium. *Chlamydomonas* cells of cw15–325 strain were grown in ammonium-containing medium (Con, control) and transferred to media containing 4 mM, 100 μM, or 10 μM NO_3_^−^ in the light for 0.5 h, 1 h, 2 h or 4 h. Values are means ± SE of three biological replicates and three technical replicates and are given as expression level relative to a house-keeping gene *RACK1* that has a value of 1.The increase in *NIT1* transcript abundance was used as a positive control (insertion). **b** Changes in nitrate levels in the media. *Chlamydomonas* cells of cw15–325 strain were grown in ammonium-containing medium and transferred to media containing 4 mM, 100 μM, or 10 μM NO_3_^−^ in the light. At the indicated times, the amounts of nitrate present in the media were quantified. ND, not determined. Values are means ± SE of three biological replicates. **c**Time course of the PII protein accumulation during incubation of illuminated cells in nitrate-containing medium. PII levels were analyzed by western blotting in the same conditions as in (**a**). Each line corresponds to 20 μg of soluble proteins extracted from samples taken from cultures at the time points indicated. Quantitation of protein blots is given as proportion of signal in test variant to control variant. HSP70B signal served as a loading control

Ammonium and nitrate are the main nitrogen sources for *Chlamydomonas* [37, 38]. There is evidence that *GLB1* transcript levels are rather low in cells grown in the presence of ammonium [21]. We wanted to investigate whether or not the expression of *GLB1* is influenced by nitrate. In cw15–325, upregulation of *GLB1* was detected in nitrate alone (Fig. 1a). Nitrate applied at concentration of 4 mM has been reported previously to induce *NIT1* gene encoding nitrate reductase [24]. In 4 mM NO_3_^−^-exposed cells, the *GLB1* transcript level was increased after 30 min and reached a maximum within 1–3 h of incubation. During further exposure to nitrate, the gene expression decreased again approximately 2.4 times higher than the control level. When *Chlamydomonas* cells were exposed to 100 μM nitrate, we observed the same expression levels as in 4 mM nitrate medium. Decreasing the nitrate concentration to 10 μM resulted in an even higher increase in the *GLB1*mRNA abundance (2.3 fold after 30 min and 4.5-fold after 1 h) (Fig. 1a). Importantly, during exposure of cw15–325 cells (*arg7–8*) to nitrate, arginine was added to the medium. Thus, the observed increase in *GLB1* transcripts was not caused by nitrogen starvation. Moreover, nitrate remaining in the media during experiment was also assayed (Fig. 1b). The data suggest that that a saturating effect observed between 4 mM and 90 μM of nitrate.

Interestingly, *GLB1* demonstrated a rather similar expression pattern to *NIT1* (Fig. 1a, insertion). However, in contrast to *GLB1*, the higher levels of *NIT1* up-regulation were reached when cells were exposed to 4 mM nitrate.

Next, we asked the question whether the nitrate-induced upregulation of *GLB1* expression is accompanied by an increase in the PII protein. Compared to cells grown in ammonium, levels of PII were indeed higher in cells incubated in nitrate (Fig. 1c). The data suggest that the PII is induced by nitrate.

### *GLB1* transcription responds to the NH_4_^+^/NO_3_^−^ balance

**Fig. 2:**
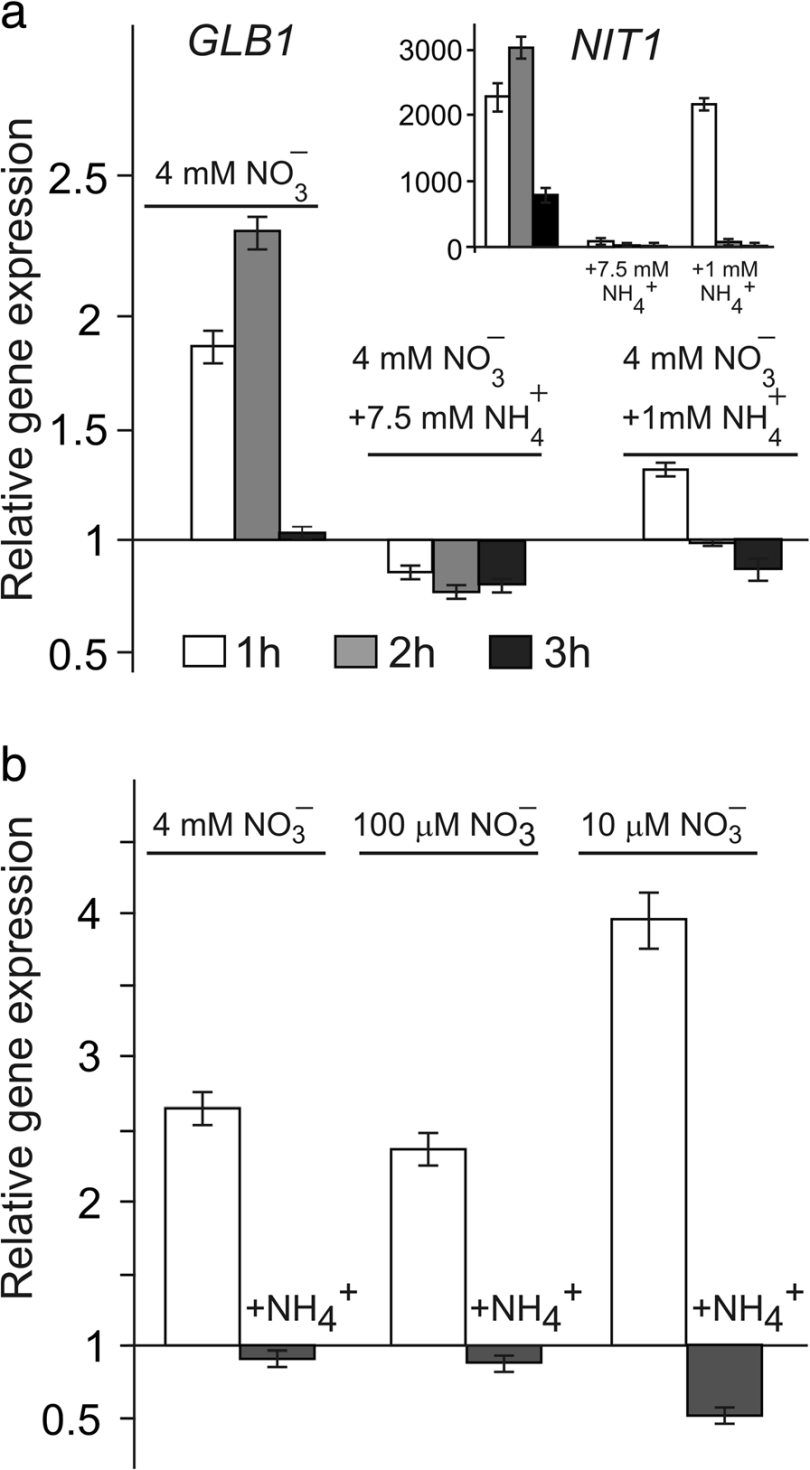
*GLB1* transcription depends on the balance of ammonium and nitrate in the medium. **a** *GLB1* transcript levels were determined in *Chlamydomonas* cells of cw15–325 strain grown in ammonium-containing medium and transferred to media containing 4 mM NO_3_^−^, 4 mM NO_3_^−^ + 7.5 mM NH_4_^+^ or 4 mM NO_3_^−^ + 1 mM NH_4_^+^. Values are means ± SE of three biological replicates and three technical replicates and are given as expression level relative to a house-keeping gene *RACK1* that has a value of 1. Expression level at 0.5 h is considered as a control. The increase in *NIT1* transcript abundance was used as a positive control (insertion). **b** *GLB1* transcript levels were determined in *Chlamydomonas* cells of cw15–325 strain grown in ammonium-containing medium and transferred to media containing the indicated concentrations of NO_3_^−^ and 1 mM NH_4_^+^ for 2 h. Values are means ± SE of three biological replicates and three technical replicates and are given as expression level relative to a house-keeping gene *RACK1* that has a value of 1

In *Chlamydomonas*, the NH_4_^+^/NO_3_^−^ balance was shown to be sensed by cells modulating expression of the nitrogen pathway genes [39]. To test whether *GLB1* transcription responds to a balance of NH_4_^+^ and NO_3_^−^, we monitored the accumulation of *GLB1* transcripts in *Chlamydomonas* cells incubated in media containing different concentrations of NH_4_^+^ (1 mM or 7.5 mM) with 4 mM NO_3_^−^. In cells supplemented with 4 mM NO_3_^−^ + 7.5 mM NH_4_^+^ *GLB1* expression was fully blocked (Fig. 2a). However, *GLB1* mRNA abundance was higher in 4 mM NO_3_^−^ + 1 mM NH_4_^+^, although significantly lower than in 4 mM NO_3_^−^ medium. Therefore, like *NIT1* (Fig. 2, insertion; [39]) *GLB1* expression responds quantitatively to extracellular NH_4_^+^. Moreover, addition of 1 mM NH_4_^+^ to media containing different concentrations of NO_3_^−^ (4 mM, 100 μM and 10 μM) caused the more severe reduction in *GLB1* transcription at the lowest nitrate concentration (Fig. 2b). These data additionally support the idea that and NH_4_^+^/NO_3_^−^ balance may play an important role in the regulation of the gene of interest.

### *GLB1* is repressed by NO

**Fig. 3:**
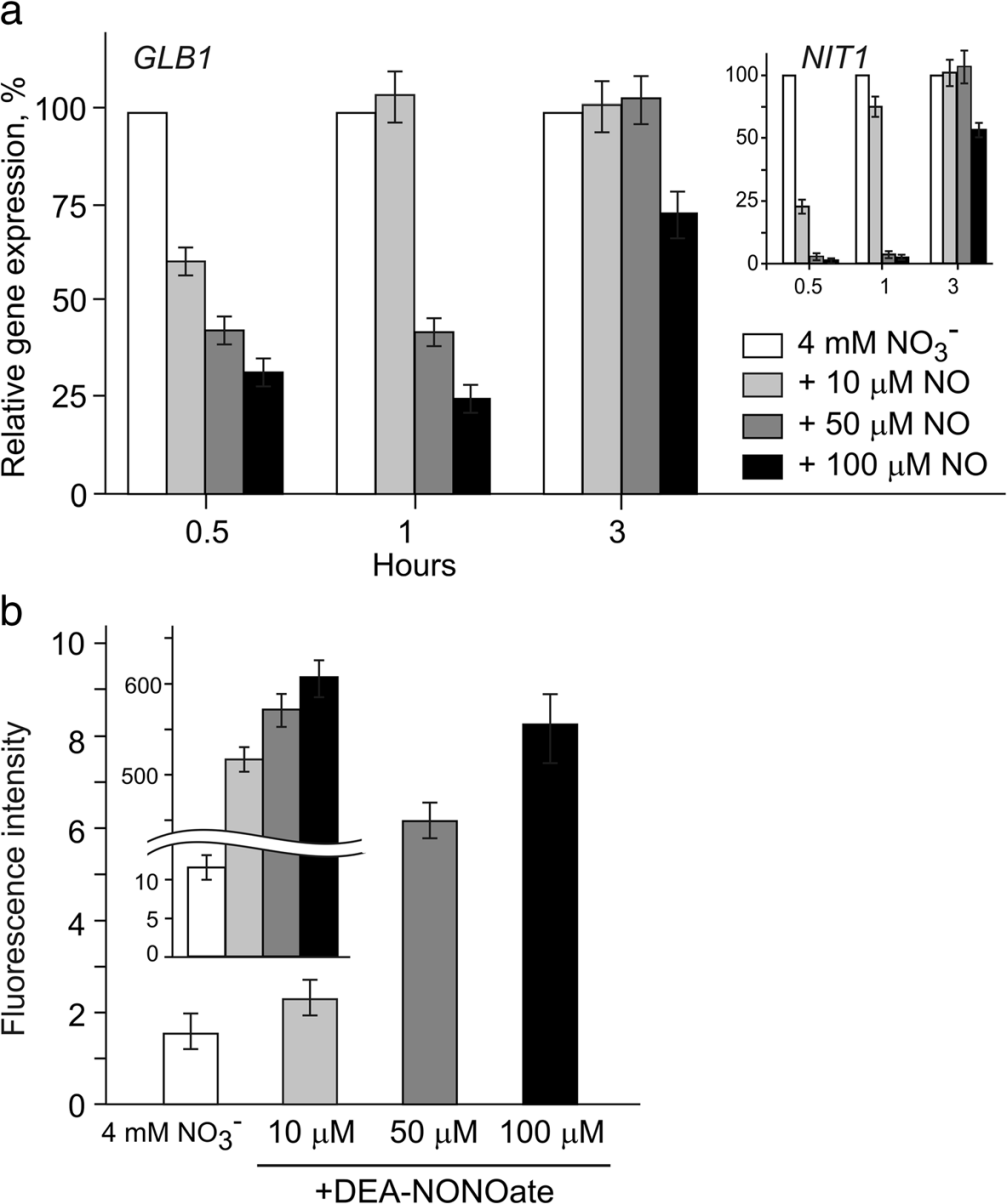
Effects of DEA-NONOate on the nitrate-induced expression of *GLB1* gene and the levels of intracellular nitric oxide. **a** The effect of DEA-NONOate (10 μM, 50 μM or 100 μM) on the *GLB1* transcripts accumulation was determined in 4 mM nitrate-induced *C. reinhardtii* cells of cw15–325 strain for 0.5 and 1 h**.** Nitrate-induced cells represent controls (set to 100%) without the added DEA-NONOate. Values are means ± SE of three biological replicates and three technical replicates and are given as expression level relative to a house-keeping gene *RACK1* that has a value of 1. The increase in *NIT1* transcript abundance was used as a positive control (insertion). **b** Fluorescence intensity due to intracellular NO was determined using 1 μM DAF-FM DA and was expressed as arbitrary units per μg protein. Cell autofluorescence was subtracted from the total fluorescence obtained. Insertion shows NO levels in the media (see Materials and Methods). Data are the means±SE from three independent experiments

Stimulation of NO generation has been reported to be dependent on the ammonium concentration in the medium and as a consequence to control the expression of the nitrogen pathway genes in *Chlamydomonas* cells [24]. The above results (Fig. 2) suggest that NO may be involved in the control of *GLB1* expression. To test this, the nitrate-induced cells were treated with DEA-NONOate as NO donor [40]. The concentrations of DEA-NONOate used (10 μM, 50 μM, and 100 μM) caused a significant inhibition of *GLB1* expression after 30 min (Fig. 3a). Interestingly, the expression of this gene was largely recovered after 1 h and 3 h in the presence of 10 μM or 50 μM and 100 μM DEA-NONOate, respectively, hinting that NO may be converted in NO_3_^−^ for this period. DEA-NONOate also exhibited a negative effect on the transcription of *NIT1* used as a control gene (Fig. 3a, insertion). These results are consistent with a balance of NO levels detected in cells and in culture broth (Fig. 3b). The combined real-time PCR and NO detection analysis suggested that mRNA levels of *GLB1* were repressed by NO. We also propose that there is subtle regulation of the NO levels which control *GLB1* expression, probably through fine tuning of the components mediating cycle NO_3_^−^ **→** NO_2_^−^ **→** NO **→**NO_3_^−^.

### Nitrite induces *GLB1* gene and NR promotes NO-dependent *GLB1* repression

**Fig. 4:**
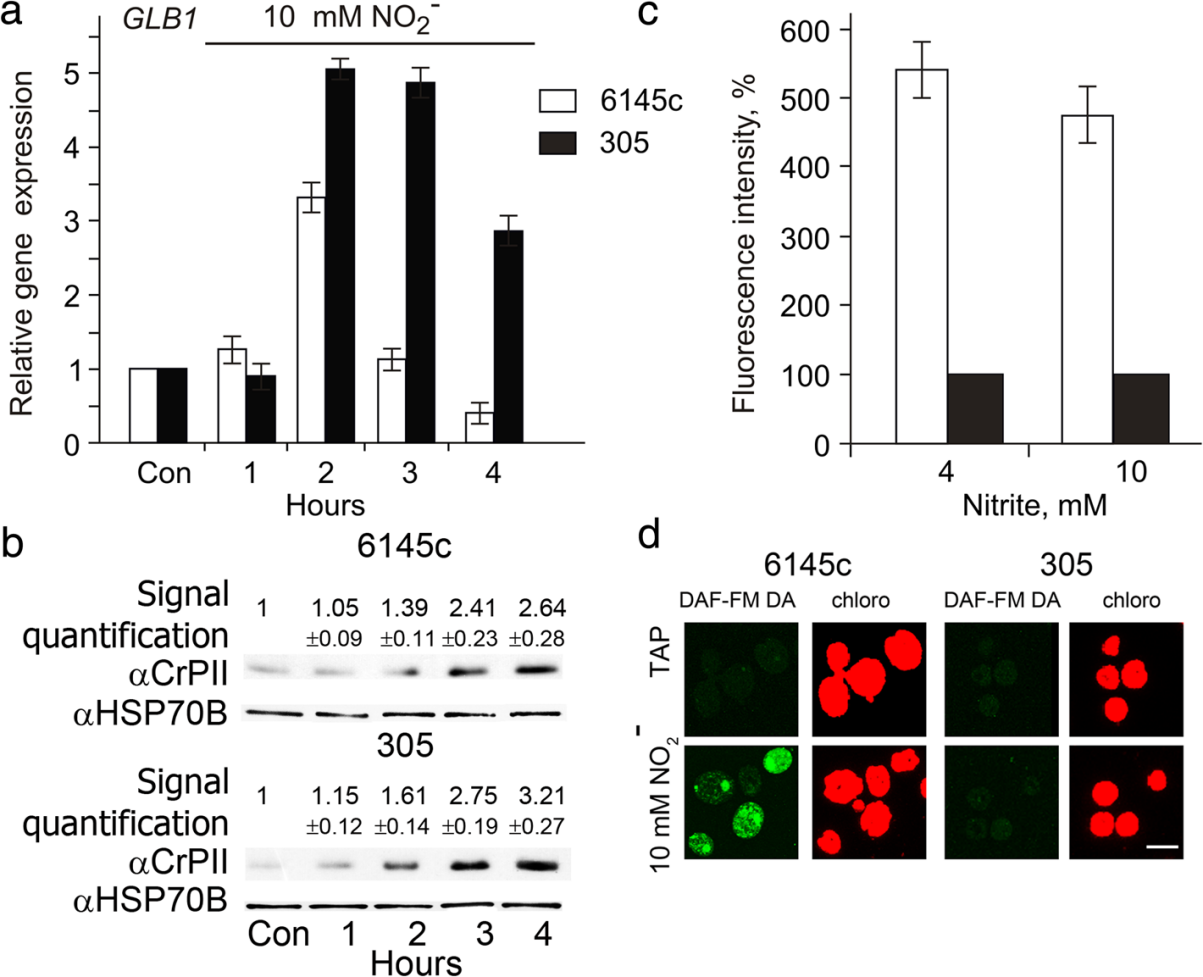
Nitrate reductase promotes NO-dependent *GLB1* repression. **a** Time course of the *GLB1* transcripts accumulation during incubation of illuminated cells in nitrite-containing medium. *Chlamydomonas* cells of the wild strain 6145c and its derivative mutant 305, affected in NAD(P) H-NR activity and without diaphorase-NR activity, were grown in ammonium-containing medium and transferred to medium containing 10 mM NO_2_^−^ in the light for 1, 2, 3 or 4 h. Values are means ± SE of three biological replicates and three technical replicates and are given as expression level relative to a house-keeping gene *RACK1* that has a value of 1. **b** Time course of the PII protein accumulation during incubation of illuminated cells of the strains 6145c and 305, in nitrite-containing medium. PII levels were analyzed by Western blotting in the same conditions as in (**a**). Each line corresponds to 10 μg of soluble proteins extracted from samples taken from cultures at the time points indicated. Quantitation of protein blots is given as proportion of signal in test variant to control variant. HSP70B signal served as a loading control. **c** NO production in nitrite-induced cells. *Chlamydomonas* cells of the strains 6145c and 305 were grown in TAP medium and transferred to nitrite-containing medium in the light for 15 min. Fluorescence intensity due to intracellular NO was determined using DAF-FM DA and was expressed as arbitrary units per μg chlorophyll . Cell autofluorescence was subtracted from the total fluorescence obtained. Fluorescence in the strain 305 is considered as control (set to 100%). Data are the means±SE from three independent experiments. Production of NO was measured by the microplate reader CLARIOstar (BMG). **d** NO visualization by confocal microscopy. Images of cells grown in TAP (TAP) or incubated in nitrite-containing medium (10 mM NO_2_^−^) for 15 min. The left-hand panels show DAF-FM fluorescence (green color) while the right-hand panels show Chl autofluorescence (red color). Scale bar equals 10 μm

In *Chlamydomonas* cells, NO appears as a consequence of nitrite accumulation [28]. To further explore the relationship between *GLB1* expression levels and NO generation, two strains were assayed: the wild strain 6145c and its derivative mutant 305, which, affected in NAD(P) H-NR activity and without diaphorase-NR activity. Both strains were incubated in 10 mM nitrite. Unexpectedly, nitrite increased *GLB1* transcript levels in cells (Fig. 4a). In addition, the *nit1* mutant showed higher levels of *GLB1* expression than WT (6145c), consistent with inability of the strain 305 to supply NAD(P)H electrons to nitrite [27].

Next, we asked the question whether the increased *GLB1* mRNA levels correlate with a change in PII protein content in nitrite-induced cells. Although the kinetics of changes in *GLB1* mRNA levels were not similar to the kinetics of changes in PII protein levels in both strains, the difference in PII protein abundance between parental strain 6145c and mutant 305 (Fig. 4b) was evident. The data suggest that *GLB1* upregulation is dependent of nitrite. This result is in agreement with the fact that the NR mutant without diaphorase-NR activity did not show significant NO signal (Fig. 4c and d). In contrast to the mutant, very strong NO fluorescence appeared in the 6145c strain, supporting a correlation between NO generation and *GLB1* mRNA abundance. Thus, these experiments allow nitrite to be added as a player in the control of *GLB1* expression together with nitrate and NO. Taken together, the results strengthen the idea that control of *GLB1* expression is regulated by a complex mechanism in which NO produced via NR/NOFNiR plays a crucial role.

### THB1 controls *GLB1* expression via the detoxification of NO

**Fig. 5:**
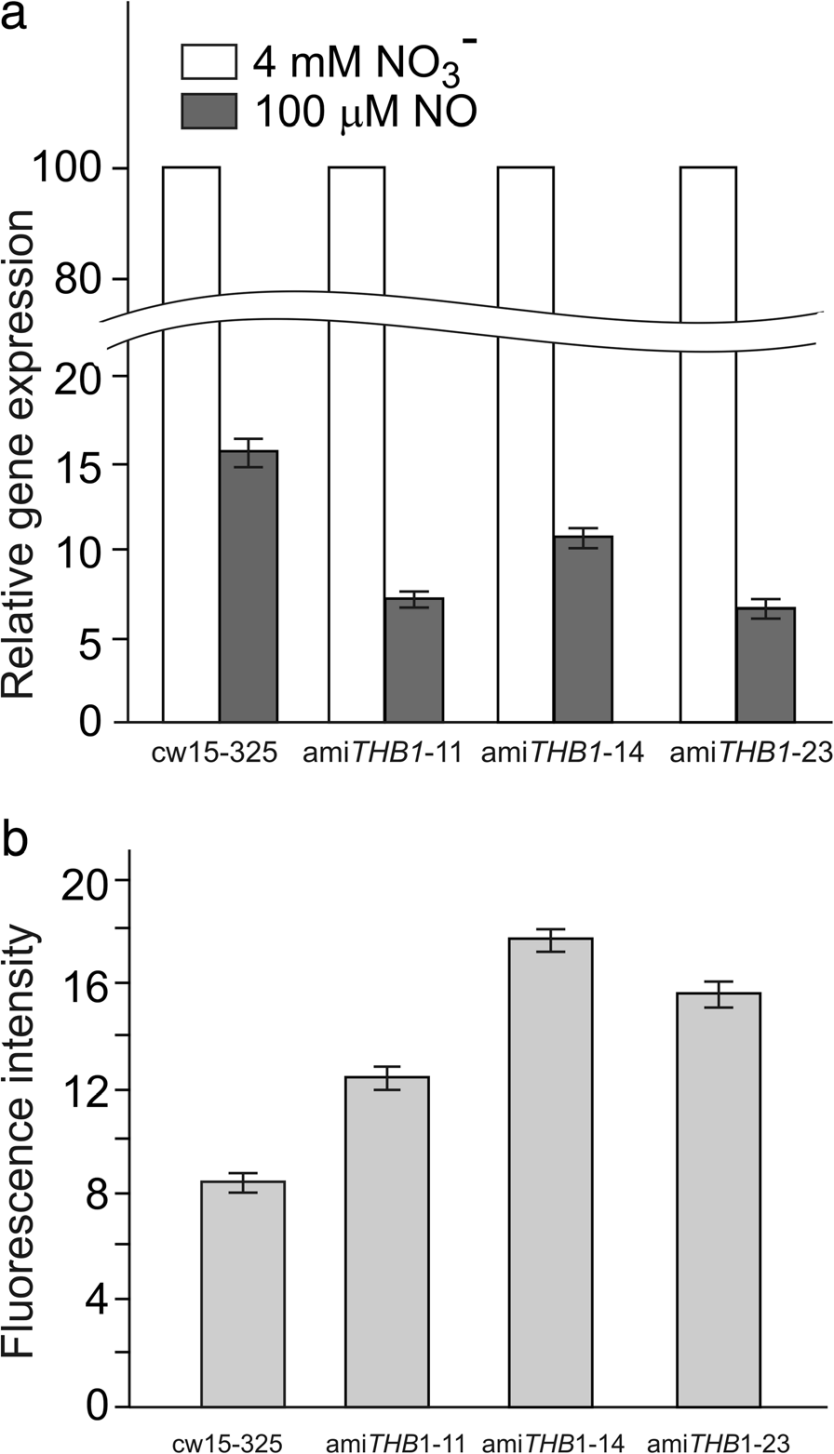
Effects of reduced THB1 levels on *GLB1* expression and NO generation. **a** Time course of the *GLB1* transcripts accumulation during incubation of cw15–325 and *ami*RNA-*THB1* cells in 4 mM nitrite-containing medium with or without DEA-NONOate (100 μM). Values are means ± SE of three biological replicates and three technical replicates and are given as expression level relative to a house-keeping gene *RACK1* that has a value of 1. **b** Fluorescence increase was measured in cw15–325 and *amiRNA-THB1* cells following the incubation in 4 mM nitrate with or without of DEA-NONOate (100 μM) for 15 min. Fluorescence intensity due to intracellular NO was determined using DAF-FM DA and was expressed as arbitrary units per μg protein. Cell autofluorescence was subtracted from the total fluorescence obtained. Data are the means±SE from three technical replicates of a representative experiment. Production of NO was measured by the microplate reader CLARIOstar (BMG)

Previously, it was shown that NR uses the truncated hemoglobin THB1 in the conversion of NO to nitrate [28]. We therefore asked whether THB1 exerts any effect on the expression of *GLB1*. To test this, the cells of cw15–325 (WT) and THB1 knock-down strains were incubated in 4 mM nitrate with or without DEA-NONOate. Nitrate-induced expression of *GLB1* was shown to be antagonized by NO (Figs. 3 and 4). As shown in Fig. 5, the downregulation of *THB1* impaired the transcription of *GLB1* in the presence of NO generator: compared with cw15–325, transcript accumulation for gene of interest in all *THB1*-*ami*RNA strains was reduced on average from 1.5-fold to 2.3-fold. These results are in agreement with the higher levels of NO measured in THB1 knock-down strains compared to parental strain (cw15–325). Collectively, these results suggest that reduction in *THB1* expression allows NO concentration to increase, triggering down-regulation of *GLB1* transcription.

### NO-dependent *GLB1* repression is released by guanylate cyclase (GC) inhibitor

**Fig. 6:**
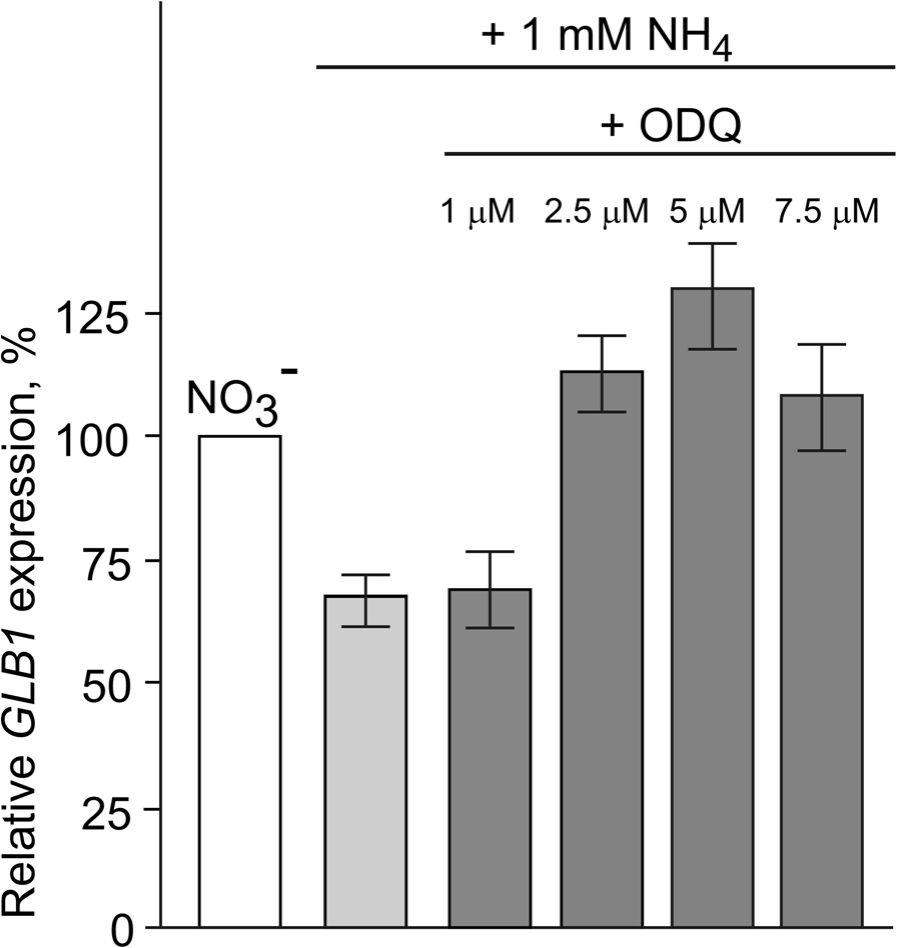
Effects of guanylate cyclase inhibitor on *GLB1* repression in medium containing nitrate and ammonium. *GLB1* transcript levels were determined in *Chlamydomonas* cells of cw15–325 strain grown in ammonium-containing medium and transferred to media containing 4 mM NO_3_^−^ + 7.5 mM NH_4_^+^ with or without 1 μM, 2.5 μM, 5 μM or 7.7 μM 1H-[1,2,4]oxadiazolo-[4,3-a]quinoxalin-1-one (ODQ). Values are means ± SE of three biological replicates and three technical replicates and are given as expression level relative to a house-keeping gene *RACK1* that has a value of 1

Our analyses show that *GLB1* is transcriptionally regulated by the same mechanisms than *NIT1* gene that is repressed by NO (Figs. 2, 3 and 4). Repression of *NIT1* by NO is mediated by NO-dependent GC activity [24]. It would thus be interesting to test the GC inhibitor in order to better decipher how the *GLB1* transcription is inhibited by NO. We found that ODQ, a selective inhibitor of NO activated GC [41], caused derepression of the *GLB1* in ammonium containing medium when applied at concentrations 2.5 μM, 5 μM or 7.5 μM, whereas 1 μM of this inhibitor had no effect (Fig. 6). This result allows us to propose that, by sensing NO, GC activity could be involved in *GLB1* repression.

## Discussion

Plant chloroplasts contain cyanobacterial-like PII homologues [6]. However, unlike PII proteins from cyanobacteria, plant PII proteins seem not to be covalently modified [20, 21]. Furthermore, in cyanobacteria, PII signaling is involved in the regulation of nitrate assimilation and gene expression through co-activator of the global nitrogen control factor NtcA [5]. Importantly, no homologues of PipX and NtcA are conserved in plants [12]. In representative plants, PII proteins are regulated at the transcriptional level [20–22]. In this study, we demonstrate that expression of *Chlamydomonas* PII is under the complex control of positive signals (i.e., nitrate and nitrite) and negative signals (NO), and *GLB1* gene expression pattern is very similar to that observed for nitrate assimilation genes.

Ammonium is the preferred nitrogen source for *Chlamydomonas.* In ammonium-containing medium *Chlamydomonas* PII is expressed at low levels [21]. Interestingly, the second major nitrogen source, nitrate, induces *GLB1* transcription (Fig. 1). The cells might use this transcription regulation to limit PII levels under optimal nutritional conditions**.** It would thus be interesting to test whether *GLB1* is strictly sensitive to ammonium or responds to a balance of ammonium and nitrate. We found that changes in the nitrate concentration modulate the response of *GLB1* gene to ammonium (Fig. 2). Together these data stress the point that, a balance of positive and negative signals regulates *GLB1*.

A part of the response to ammonium/nitrate balance is a change in the intracellular concentration of NO [39]. We have shown herein that NO represses expression of *GLB1* (Fig. 3). Interestingly, *GLB1* exhibits a similar transcription pattern to *NIT1* (Fig. 3a, insertion) and other genes from the nitrate assimilation cluster [24, 38, 42, 43], suggesting that the expression of PII is tightly controlled with regard to the nitrogen source and the physiological state of cells. Physiological studies of *Arabidopsis* suggested a role of PII in nitrite uptake [44]. In addition, *GLB1* expression is up-regulated in the presence of nitrite (Fig. 4a). Together, these observations allow us to speculate that PII protein may also play some uncharacterized roles in control of nitrogen assimilation in *Chlamydomonas* cells.

In the cytoplasm, nitrite is converted into NO by NR that is partnered with NOFNiR [27]. Thus, NR modulates both the levels of NO and the amounts of nitrite available for metabolism. Importantly, the diaphorase-NR activity is required for supplying NAD(P)H electrons to nitrite [27]. We propose that *GLB1* transcription is dependent on the dual system of NR and NOFNiR through fine tuning of NO levels. The fact that the diaphorase-NR activity is required to repress PII levels in the presence of nitrite (Fig. 4a; b) supports this idea. In agreement with these data, spectrofluorometric assays (Fig. 4c) and confocal microscopy (Fig. 4d) with DAF-FM DA allowed us to detect higher fluorescence levels in parental strain 6145c than *nit1* mutant. More generally we could propose that nitrite-dependent NO production plays role in the control of PII expression dynamics, ensuring possible interconnecting/coordinating PII-dependent and nitrate assimilation pathways.

As NO is toxic, plants have protective mechanisms to defend themselves. Like higher plants, *Chlamydomonas* cells use hemoglobins to convert NO into nitrate [28, 45, 46]. It has been previously shown that a truncated hemoglobin 1, THB1, has NO-dioxygenase activity [28]. In *amiTHB1* strains, the nitrite-responsive accumulation of *GLB1* transcripts is impaired (Fig. 5a). As expected, DEA-NONOate in nitrite-containing medium resulted to higher fluorescence levels in *THB1*-knockdown transformants than in parental strain (Fig. 5b). Taken together, these results strengthen the notion that NO acts as a signaling molecule for the transcriptional regulation of *GLB1* gene, and THB1 is involved in this NO-dependent pathway.

**Fig. 7:**
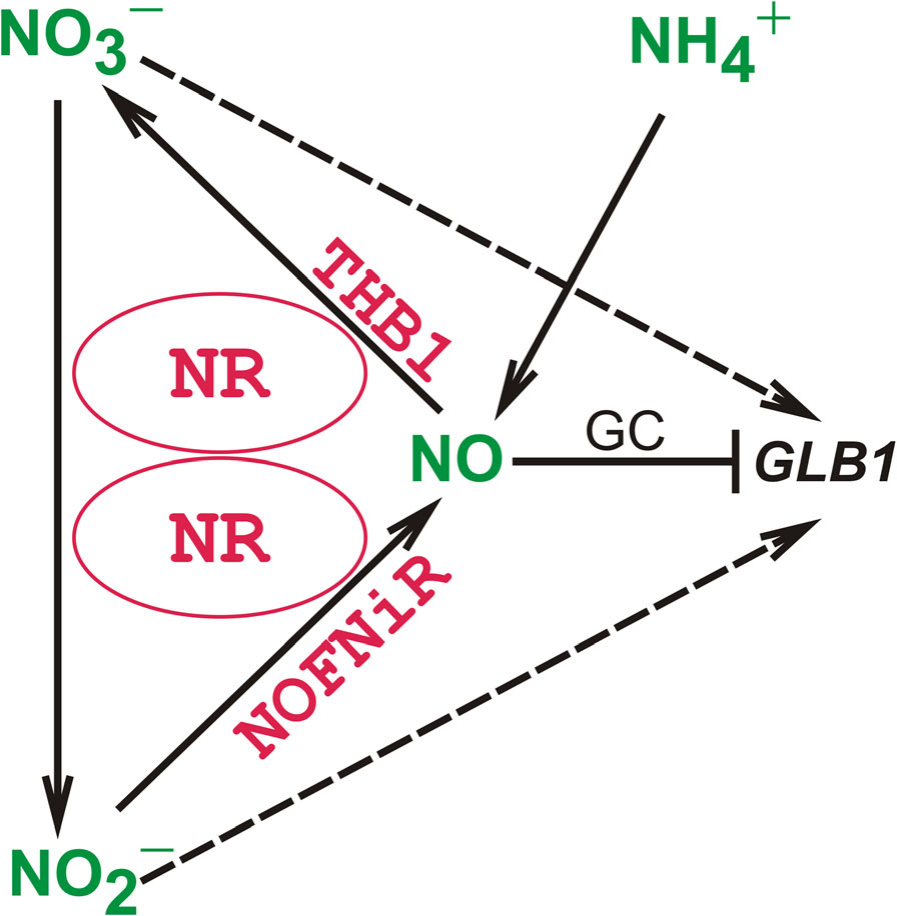
Model for a nitrogen-dependent control of *GLB1* expression. Nitrate and nitrite act as positive regulators of *GLB1* transcription (dotted arrows). Conversely, NO that is mediated via the components of nitric oxide cycle or via a rise in intracellular ammonium, represses *GLB1* transcription (thick T-like line). GC may also control *GLB1* expression in NO-GC-dependent manner

The mechanism implicated in the repression of *GLB1* by NO is not clear, although in *Chlamydomonas*, six proteins CYG11, CYG12, CYG15, CYG38, CYG56 and CYG57 share the same domains structure with mammalian NO sensors, the soluble guanylate cyclases [30, 47–49]. In animals, the GCs activity is usually stimulated by the binding of NO to their heme group [50]. Moreover, CYG56 participates in ammonium-mediated *NIA1, NRT2.1, AMT1.1* and *AMT1.2* repression through a pathway that involves NO and cGMP [24, 49]. Importantly, inhibition of the NO-dependent GC by ODQ releases cells from ammonium repression of not only genes from the nitrate assimilation cluster but also of *GLB1* gene (Fig. 6). This correlation emphasizes that regulation of nitrate assimilation genes and *GLB1* are closely related and support the hypothesis that a soluble guanilate cyclase may also control *GLB1* expression in NO-GC-dependent manner. Interestingly, only CYG56 is up-regulated in ammonium compared with nitrate-containing medium [24], hinting that ammonium-mediated repression of *GLB1* might be regulated through CYG56-dependent signaling. However, which guanylate cyclase is a pivotal factor for the regulation of *GLB1* in ammonium-containing medium has to be analyzed further. The experiments described here establish ammonium as a component of the signaling pathway for the negative control of the *GLB1* transcription. However, they do not address how ammonium acts to repress *GLB1* gene. Moreover, we cannot rule out that NO-dependent *GLB1* repression by nitrite and ammonium may go through the various routes. We propose a model (Fig. 7) in which PII expression is under complex control of positive and negative signals depending on nitrogen source.

## Conclusions

Our main conclusion is that PII level is tightly controlled with regard to the nitrogen source and the physiological state of cells. We provide evidence on that NO via the components of nitric oxide cycle is involved in the negative control of *GLB1*. On the other hand, nitrate and nitrite induce this gene transcription. Therefore, important regulatory layer in the PII-dependent signal transduction system in *Chlamydomonas* could be that the concentration of the PII protein must be balanced in order for the signaling mechanism to function properly – the system is fine-tuned.

## Additional files

Additional file 1: **Table S1.** Primers sequence that were used for real time PCR analysis. (XLSX 10 kb)

## Abbreviations

DAF-FM DA: 4-amino-5-methylamino-2′7’-difluorofluorescein diacetate
DEA-NONOate: 2-(*N*,*N-*diethylamino)-diazenolate 2-oxide sodium salt
GC: Guanylate cyclase
NO: Nitric oxide
NOFNiR: Nitric oxide-forming nitrite reductase
NR: Nitrate reductase
ODQ: 1H-(1,2,4)oxadiazolo(4,3-a) quinoxalin-1-one
TAP: Tris-acetate-phosphate medium
THB1: Truncated hemoglobin 1
